# Workplace Social Support as a Mediating Factor in the Association between Occupational Stressors and Job Burnout: A Study in the Taiwanese Nursing Context

**DOI:** 10.1155/2023/5599128

**Published:** 2023-08-29

**Authors:** Li-Chu Wu, Chia-Yi Chou, Chi-Ya Kao

**Affiliations:** ^1^Department of Nursing, Kaohsiung Veterans General Hospital, Kaohsiung 81346, Taiwan; ^2^School of Shu-Zen Junior College Medicine and Management, Kaohsiung 82144, Taiwan; ^3^Department of Environmental Science and Occupational Safety and Hygiene, Tajen University, Pingtung County 90741, Taiwan

## Abstract

**Background:**

Nurses confront high-stress, high-stakes work environments due to evolving disease patterns and growing healthcare needs. The nurse-patient ratio in Taiwan is higher than in other countries, necessitating effective strategies to mitigate nurse burnout and enhance the quality of patient care.

**Design:**

A cross-sectional study design was employed.

**Methods:**

From January to April 2019, 500 nurses were recruited from a medical center in Kaohsiung City, southern Taiwan. Participants completed a questionnaire addressing workplace social support, stressors faced by nurses, and job burnout. Data were analyzed using descriptive statistics, one-way analysis of variance, *t*-test evaluations, Pearson's correlation analyses, and a structural equation model with maximum likelihood estimation.

**Results:**

The findings revealed that a portion of nurses experienced high rates of personal burnout (7.20%), work-related burnout (5.00%), and client-related burnout (4.80%). The relationships among workplace social support, nurses' stressors, and job burnout were all substantial, exhibiting correlation coefficients ranging from −0.318 to 0.828. The direct effect of nurse stress on job burnout was 0.551, comprising 90.7% of the cumulative effect. In contrast, the indirect effect of nurse stress on job burnout, considering workplace social support, amounted to 9.3% of the total effect, with a value of 0.056.

**Conclusions:**

The study underscored the importance of addressing job burnout among nurses in Taiwan. Workplace social support may function as a mediating factor in the relationship between nurses' stressors and job burnout. *Implications for Nursing Management*. The results suggest that healthcare administrators should prioritize workplace social support initiatives. These efforts could help identify and address nurses' stressors, promote work-life balance, and reduce nurse-patient ratios and work overload.

## 1. Introduction

Nurse burnout is a prevailing global concern within the healthcare sector, presenting significant implications for patient care and the well-being of nurses. Despite growing awareness and research in this area, a knowledge gap persists regarding the complex factors contributing to this phenomenon, particularly in diverse cultural and socio-economic contexts. This study explores a unique aspect of the nursing field by investigating the relationship between workplace social support (WSS), job stress, and nurse burnout, specifically in the Taiwanese healthcare setting. Although nurse burnout is a widely researched topic, the distinct emphasis on the role of WSS in this context provides new and unique information. The unique aspects of the Taiwanese healthcare environment, including its rapidly aging population and changing disease trends, further underscore the distinctiveness of this research.

From a theoretical perspective, there is a recognized need to deepen our understanding of how different elements interact to exacerbate or mitigate nurse burnout. While previous studies have illuminated the individual impacts of job stress and social support on nurse burnout [1], the interplay between these factors, especially the mediating role of WSS, remains relatively underexplored. This gap in the existing literature suggests a missed opportunity to develop a comprehensive theoretical framework that could more accurately depict the factors contributing to nurse burnout. On a practical level, the high rates of nurse burnout are indicative of significant challenges within the nursing profession and healthcare institutions. Despite various attempts to address this issue, we still lack comprehensive strategies that take into account all aspects of nurse burnout, including the role of social support in the workplace. This suggests a crucial gap between current practice and potential strategies to mitigate nurse burnout.

This study aims to bridge these theoretical and practical gaps. By using structural equation modelling (SEM) to investigate the mediating role of WSS in the relationship between job stress and nurse burnout, we seek to enrich the theoretical understanding of these constructs. Simultaneously, the findings of this research aim to provide practical insights that can assist healthcare administrators and nurse managers in developing effective strategies to mitigate nurse burnout, thereby improving the quality of patient care and nurses' well-being.

## 2. Background

Research in Taiwan suggests that nurses experience higher rates of work-related stress than other medical professionals [2, 3]. Healthcare professionals play a crucial role in delivering high-quality healthcare. Unfortunately, job burnout has emerged as a significant concern in recent years, adversely affecting personal health and job satisfaction [4]. Occupational stress is a major contributor to job burnout among nurses and other populations [5]. Increased levels of work stress have been found to contribute to job burnout among healthcare professionals, including nurses [6]. The impact of stressful events on individuals is determined by their perception of stress [7]. Consequently, effective management of work stress is critical for preventing negative work attitudes and maintaining high-quality nursing care.

The intensity of occupational stress has been proposed as a factor contributing to job burnout in various populations [5], including healthcare workers who are prone to job burnout due to their high-stress work environments [6]. A shortage of nursing professionals and long-term care personnel in Taiwan has led to a higher nurse-patient ratio significantly higher than that in other countries [8–10]. According to data published by Taiwan's Ministry of Health and Welfare, the global average nurse-to-patient ratio in medical centers is 1 : 7.7, while in Taiwan, it is 1 : 8.6 [10, 11]. Various factors influence the nursing workforce shortage, including ineffective policies, inadequate planning and recruitment, and a lack of leadership management [12]. Nursing turnover is a complex issue impacted by numerous factors, including insufficient professional recognition of nurses, inadequate social support, heavy workloads, and low job satisfaction. An increased patient load can reduce nurses' perception of social support in the workplace, thereby negatively affecting their job satisfaction and performance. Furthermore, recent studies have demonstrated that occupational stress can directly lead to job burnout and indirectly influence it through other factors, such as social support, as suggested by some researchers [13].

In light of these concerns, nurse burnout, exacerbated by high work-related stress and occupational hazards, has become a critical issue in Taiwan's healthcare sector. This problem not only affects the well-being of nurses but can also lower job satisfaction, increase turnover rates, and potentially compromise the quality of patient care.

The relationship between job stress, WSS, and job burnout can be explained by both the job demands-resources (JD-R) model [14, 15] and the buffering effect model of social support [16, 17]. Researchers have also proposed that social support can serve as a buffer against the detrimental effects of stressful events on individuals' physical and mental health [18]. Yeh et al. [19] discovered that emotional exhaustion and work-family conflict (WFC) mediate the relationship between nurse burnout and relevant outcomes. These findings offer valuable theoretical and practical implications for organizations aiming to implement interventions to reduce burnout among nursing personnel [19]. According to the JD-R model, WSS can decrease perceived stress and prevent burnout in nursing professionals. Job demands, such as work overload and occupational hazards, can contribute to job burnout. However, social support can offer emotional and informational assistance, reduce isolation, and enhance control over the work environment. The buffering effect model suggests that social support can serve as a safeguard against the adverse effects of stress. Workplace stressors, such as work overload and occupational hazards, can cause perceived stress, but high levels of WSS, including support from supervisors, coworkers, and the organization, can counteract these stressors by providing emotional and informational support, fostering a sense of belonging, and bolstering self-esteem. This, in turn, can lead to lower levels of perceived stress.

**Figure 1:**
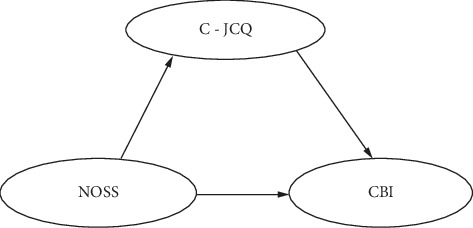
The proposed role of workplace social support (C-JCQ) as a mediator in the relationship between occupational stressors (NOSS) and job burnout (CBI).

Based on the JD-R model and the buffering effect model of social support, we aim to (a) determine the prevalence of job burnout among nursing professionals, (b) investigate the relationship between stress, WSS, and job burnout among nurses, and (c) examine the mediating role of WSS in the association between occupational stress and job burnout. Consequently, we propose the following hypotheses: (a) there will be a positive correlation between job burnout and occupational stress; (b) WSS will be negatively correlated with job burnout; and (c) WSS will moderate the positive relationship between occupational stress and job burnout. The formulated hypotheses serve as the foundation for the conceptual framework depicted in Figure 1. The figure presents three main variables: occupational stress (NOSS), WSS (C-JCQ), and job burnout (CBI). The arrows represent the hypothesized relationships between these variables. Specifically, NOSS is expected to have a positive relationship with CBI, while C-JCQ is anticipated to have a negative correlation with CBI. Furthermore, C-JCQ is hypothesized to mediate the relationship between NOSS and CBI.

## 3. Methods

### 3.1. Study Design

In this research, we adopted a cross-sectional design with the aim of answering the following research questions:

1. What is the current prevalence of job burnout among nurses in Taiwan?
2. Is there a significant relationship between occupational stress, WSS, and job burnout in the Taiwanese nursing context?
3. Does WSS play a mediating role in the association between occupational stress and job burnout among the nursing workforce in Taiwan?

### 3.2. Setting and Participants

The research included nurses employed at a public medical center in Taiwan. The inclusion criteria for the participants were as follows: (1) they had to have been employed at the institution for at least three months, (2) they could not hold administrative positions, and (3) they need to willingly agree to participate and cooperate after receiving a detailed explanation from the research team. Conversely, the study excluded: (1) specialist nurses, (2) staff in management roles, (3) nurses from nonclinical units such as the physical examination center and the aesthetic medical center, (4) staff on unpaid leave, and (5) those who did not agree to participate in the study after the explanation by the researchers. In the context of SEM, the recommended sample size for a survey is typically 10 to 15 times the number of items in the questionnaire [20]. This study included a total of 46 items (15 related to nurses' stress, 15 to workplace social support, and 16 to nurse burnout). Accordingly, to fulfil the SEM analysis requirements and enhance the reliability and validity of our findings, we need to gather at least 460 completed and valid questionnaires. These questionnaires provided demographic information and captured details about the nurses' perceived stress levels, WSS, and levels of burnout.

For this study, we collected data using a structured questionnaire distributed among nurses at a public medical center in Taiwan. The questionnaire was administered between January and April 2019, employing convenience sampling with the support of trained research assistants. Out of the 560 questionnaires disseminated, 516 were returned, yielding a response rate of 92.1%. After discarding questionnaires that were not completely filled out, we obtained a final tally of 500 valid responses from participants who completed the entire questionnaire. This sample size helps safeguard the integrity of our results and facilitates a more accurate interpretation and generalization of the data.

### 3.3. Questionnaire and Measurements

#### 3.3.1. Nurse Burnout

In this study, job burnout was measured using the Copenhagen Burnout Inventory (CBI) [21] and the Chinese version of the Occupational Burnout Inventory, which was developed based on the effort-reward imbalance model [22–24]. The self-administered questionnaire included three subscales: personal burnout (5 items), work-related burnout (5 items), and client-related burnout (6 items). Participants rated their responses on a 5-point Likert scale, ranging from 1 (never) to 5 (always). In this study, the scale's Cronbach's *α* coefficient was 0.942, with the coefficients for the three subscales being 0.904, 0.884, and 0.915, respectively. Each subscale score ranged from 0 to 100, with higher scores indicating higher levels of burnout. In this study, we defined high personal/work-related burnout as a personal/work-related burnout score of ≥80 points and high client-related burnout as a client-related burnout score of ≥65 points [25].

#### 3.3.2. Social Support

We used the Chinese version of the Job Content Questionnaire (C-JCQ) [26, 27] to assess psychosocial work characteristics and understand job stress issues. The C-JCQ consists of three subscales with 15 items. In this study, the scale's Cronbach's *α* coefficient was 0.936, and the coefficients for each subscale were as follows: workplace justice (0.918, 7 items), supervisor support (0.923, 4 items), and coworker support (0.909, 4 items), indicating acceptable internal consistencies. Participants rated each item on a Likert scale of 1 to 4 (1 = strongly disagree; 2 = disagree; 3 = agree; and 4 = strongly agree). Each subscale score ranged from 0 to 100, with higher scores indicating better workplace justice, social support, and coworker support.

#### 3.3.3. Perceived Stress

We used the Chinese version of the Nurses' Occupational Stressor Scale (NOSS) [25, 28] to measure the participants' occupational stressors. The NOSS consists of three subscales with 15 items. In this study, the scale's Cronbach's *α* coefficient was 0.942, and the coefficients for each subscale were as follows: occupational hazards (0.752, 5 items), work-life conflict (0.832, 5 items), and overload (0.772, 5 items). Participants rated each item on a Likert scale ranging from 1 (strongly disagree) to 4 (strongly agree). Each subscale score ranged from 0 to 100, with higher scores in the three dimensions of occupational hazards, work-life conflict, and overload, indicating higher levels of nurses' stress.

### 3.4. Ethical Considerations

Throughout the study, we adhered to the bioethical principles outlined in the Declaration of Helsinki. The Institutional Review Board for Medical Research Ethics at Kaohsiung Veterans Hospital (VGHKS19-CT1-05) granted approval for this study. We ensured the inclusion of all participants and preserved the anonymity and confidentiality of the collected data to protect the individuals' privacy.

### 3.5. Data Analysis

The cross-sectional nature of our study inherently carries a potential risk of common method variance (CMV) bias [29, 30]. To evaluate the presence of this bias, we used Harman's single-factor test, a commonly accepted method for this purpose [29, 31]. According to this test, if common method bias is present, a significant part of the variance should be explained by the first factor, or a single factor should emerge from the analysis. For our analysis, we subjected 46 items (15 items each on nurses' stress and workplace social support, and 16 items on nurse burnout) to an exploratory factor analysis. This was carried out using principal component analysis with varimax rotation, while forcing the extraction of a single factor. The first factor accounted for 29.61% of the variance, which falls below the 50% cut-off point. Therefore, based on these results, our study did not detect the existence of a CMV bias.

We used descriptive statistical methods to examine the demographic characteristics of the participants, including aspects such as gender, age, marital status, education level, working unit, and length of professional experience. One-way analysis of variance (ANOVA) was employed to contrast the research variables across age, unit, and work experience groups, while *t*-tests were conducted to investigate differences between groups based on gender, marital status, and educational qualifications. We also employed Pearson's correlation analyses to scrutinize the bivariate associations among the three variables: nurses' stress, WSS, and nurse burnout.

In the structural equation model (SEM) analysis, we employed the maximum likelihood estimation method by using the IBM SPSS software and AMOS 23.0. This method provides unbiased, consistent, and efficient estimates when the data are normally distributed. Before conducting the SEM analysis, we ensured that the data met the necessary assumptions, including linearity, multivariate normality, and the absence of multicollinearity. We checked for linearity by examining scatterplots, assessed multivariate normality by checking skewness and kurtosis values, and checked for multicollinearity by examining the variance inflation factor (VIF).

We evaluated the SEM using various goodness-of-fit indices. These indices included the chi-square/degrees of freedom ratio (*χ*^2^/df), the standardized root-mean-square residual (SRMR), the root mean square error of approximation (RMSEA), the goodness of fit index (GFI), the comparative fit index (CFI), and the Tucker–Lewis index (TLI). The *χ*^2^/df ratio, which measures how well the data fits the model, indicates a good fit with values under 5.0. The SRMR represents the average discrepancy between the data and the model; values up to 0.08 are acceptable. The RMSEA measures the approximation error, with values under 0.08 considered reasonable. The GFI measures how much of the variance is explained by the model, with values over 0.90 indicating a good fit. The CFI compares the fit of our model to a basic model, with values over 0.90 considered good. Lastly, the TLI, also known as the Non-Normed Fit Index, measures relative fit, with values over 0.90 indicating a good fit.

## 4. Results

Table 1 presents the demographic characteristics of the 500 participating individuals and the distribution of job burnout dimensions across categorical factors. The study population primarily consisted of female nurses (96.2%) aged between 20 and 58 years, with a mean age of 34.66 and a standard deviation of 9.85. There were no significant differences in the mean scores of personal burnout, work-related burnout, and client-related burnout across gender, age, and education groups. Approximately 35% of the participants were married, while 90% had earned a university degree or higher. Approximately 25.6% of the nurses worked in the medical ward, while 22.4% were from the surgical ward.  Table 2 expands on the demographic details, emphasizing the range of professional experience among the study participants, with a majority (53.4%) having up to 5 years of experience, 14.2% having worked between 6 and 10 years, and 32.4% boasting over 11 years of experience. Concurrently, the study highlighted the notable presence of job stress, which was observed to be at an upper-middle level. Moreover, the study revealed the prevalence of burnout to be around a mild to moderate level, with some nurses experiencing extreme burnout. Specifically, 7.20% reported high levels of personal burnout, 5.00% experienced high levels of work-related burnout, and 4.80% suffered from high levels of client-related burnout, indicating a widespread presence of severe burnout among the nurses.

Table 3 presents the results of Pearson's correlation analyses (employing a two-tailed method) among the NOSS subscales (occupational hazards, work/life conflict, overload), the C-JCQ subscales (workplace justice, supervisor support, coworker support), and the CBI subscales (personal burnout, work-related burnout, and client-related burnout). All correlations among variables were statistically significant, with coefficients ranging from −0.318 to 0.828 (*p* < 0.001). The C-JCQ was negatively related to each of the CBI subscales, including personal burnout, work-related burnout, and client-related burnout (*p* < 0.001).

**Figure 2:**
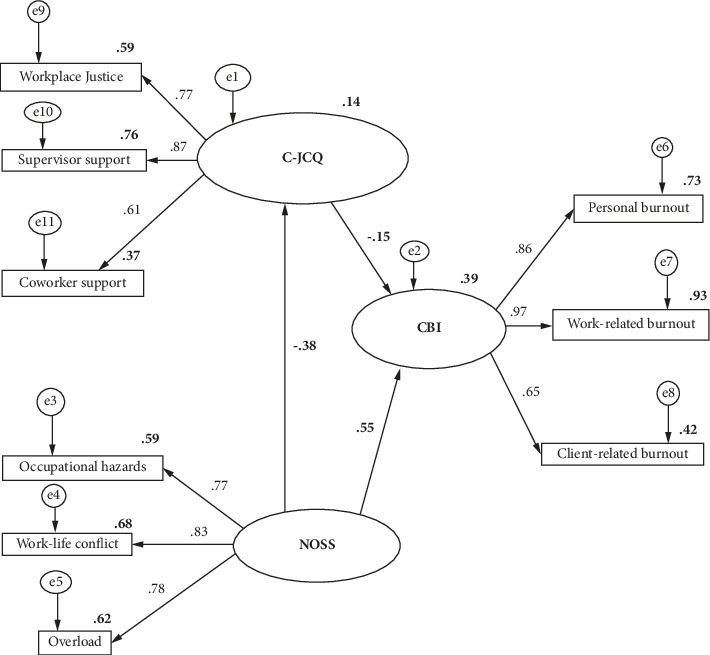
The final model and the standardized path model displayed standardized path coefficients, all of which were significant at *p* < 0.01. The C-JCQ, NOSS, and CBI were used to measure workplace social support, occupational stressors, and job burnout, respectively. C-JCQ: workplace social support, NOSS: nurses' stresses, CBI: nurse burnout, WJ: workplace justice, CS: coworker support, SS: supervisor support, OH: occupational hazards, WLC: work-life conflict, PB: personal burnout, WRB: work-related burnout, and CRB: client-related burnout.

The SEM was employed to analyze the connections among the research variables and evaluate the proposed hypotheses. Utilizing the maximum likelihood method, we adjusted the data and refined the theoretical model based on model fit indices, considering factors such as gender, age, education level, and length of work experience. The goodness of fit results for this model were as follows: *χ*^2^/df = 2.628 (less than 5.0), SRMR = 0.031 (less than or equal to 0.08), RMSEA = 0.057 (less than 0.08), GFI = 0.974 (greater than 0.90), CFI = 0.982 (greater than 0.90), and TLI = 0.973 (greater than 0.90). All these indices met their respective reference criteria, indicating that the model provided a good fit for the data [32].  Figure 2 presents the final model, which incorporates the correlations and impact pathways of the research variables, thereby confirming the study's hypotheses.

Table 4 shows the path coefficient results of the SEM. We found that the NOSS was positively related to the CBI (*β* = 0.55, *p* < 0.001) but negatively correlated with the C-JCQ of WSS (*β* = −0.38, *p* < 0.001). The C-JCQ was negatively related to the CBI (*β* = −0.15, *p* < 0.001). Among all the dimensions of NOSS, C-JCQ, and CBI, the highest absolute values were found for supervisor support (0.87), work-life conflict (0.83), and work-related burnout (0.97), respectively.

Table 5 demonstrates the direct and indirect connections between the C-JCQ and the CBI. None of the confidence intervals for each pathway include zero, indicating the significance of these relationships. The NOSS exhibited total and direct effects of 0.61 and 0.55 on the CBI, respectively. The proportion of the direct effect to the total effect of NOSS on CBI was 90.7%. The C-JCQ demonstrated an indirect effect of 0.06, which accounted for 9.3% of the total effect.

In summary, the results showed significant relationships among the variables in the study, with the C-JCQ, representing WSS, negatively correlated with both NOSS and CBI. The SEM analysis substantiated the hypothesized mediation role of WSS in the relationship between nurses' stress and job burnout. The strongest relationships were observed between supervisor support, work-life conflict, and work-related burnout.

## 5. Discussion

This study, which explores the connection between WSS and nurse burnout in Taiwan, revealed three important findings. Firstly, job burnout among Taiwanese nurses requires special attention. Second, the stressors nurses face and the social support they receive at work are related to job burnout. Lastly, WSS could potentially serve as a mediator in the association between nurses' stressors and job burnout. The study discovered that WSS positively impacts the reduction of nurses' stressors and job burnout, consistent with prior research [33]. Good organizational support, positive manager relationships, and effective care can improve the practice of nurse practitioners [34]. In addition, research indicates that higher levels of organizational support are linked to greater job satisfaction among nurses, and positive manager relationships correlate with increased job satisfaction for nurses, contributing to the overall strengthening of nurse practitioner practice.

As Taiwan undergoes rapid progress, nurses are faced with high-intensity, demanding work environments, driven by an aging population and changes in disease trends. The increase in healthcare needs and patient numbers requires nurses to develop advanced skills and dedicate more time to their clinical responsibilities. This increased workload can lead to emotional exhaustion and a decrease in personal satisfaction. Given that job burnout within healthcare settings can adversely impact the quality of patient care, examining the burnout levels among this group can enhance awareness of the challenges encountered by caregiving professionals.

The major stressors for nurses include workload, the nature of nursing work, family responsibilities, personal expectations, interpersonal relationships, and patient contact [33]. Previous studies [35, 36] have found that WSS has a positive effect on reducing nurses' stressors and a negative impact on job burnout. WSS can serve as a mediating factor in reducing perceived stress and subsequently decreasing the risk of job burnout in nursing. By enhancing social support and reducing occupational stress, healthcare managers can create a positive and supportive work environment for medical staff. This could result in decreased stress levels and burnout prevention, as perceived stress has both a direct and an indirect effect on job burnout, with the latter being mediated by social support [18].

This research examines the interconnections between nurses' stress, job burnout, and WSS, focusing on WSS's mediating role. The SEM findings revealed that nurses' stress had both direct and indirect effects on job burnout, with WSS acting as a mediator. As a mutable, state-like positive psychological resource, WSS was investigated as a mediating variable in this study. The findings suggested that nurses experiencing higher stress levels were more likely to endure physical and psychological fatigue, which could ultimately lead to an increase in their job burnout levels. Results from a previous study suggest that supervisors can help control potential stressors and mitigate burnout among nurses by supporting work-family balance. Hospitals can provide family health measures, while supervisors can facilitate a suitable working environment and promote team-building activities to enhance staff rapport [19].

Nurses often encounter workplace bullying, which can negatively affect their health, safety, and job performance. A recent study found that workplace bullying partially mediates the relationship between occupational burnout and nurses' intentions to leave their jobs [37]. Moreover, work-life conflict contributes to job dissatisfaction, which in turn increases the likelihood of nurses leaving their jobs. In contrast, WSS can reduce job dissatisfaction and turnover intentions by alleviating feelings of stress and burnout [38]. Occupational hazards encompass physical, chemical, biological, and psychosocial risks present in the workplace. With higher nurse-patient ratios, there is an increase in workload and longer working hours, leading to both physical and emotional exhaustion and a higher risk of occupational hazards. Work-life conflict arises from the mismatch between work demands and personal responsibilities. Higher nurse-patient ratios can cause longer working hours and an increased workload, leading to an imbalance between work and personal life and heightened stress levels. Work overload refers to excessive workload, responsibilities, and time pressure. With higher nurse-patient ratios, there is an increase in workload and longer working hours, resulting in feelings of being overwhelmed and stressed.

A recent study conducted in Taiwan found that work-related burnout and patient-related burnout negatively impacted job satisfaction [39]. Personal burnout may stem from various factors, including a lack of control over work, low job satisfaction, and an imbalance between work and personal life. The increase in nurse-patient ratios leads to a heightened workload and added responsibilities, resulting in a sense of being overwhelmed and increased stress. Work-related burnout can result from repetitive work, limited autonomy, and uncertain job security. The heavier workload and longer working hours brought on by higher nurse-patient ratios can cause feelings of overwork and exhaustion. Client-related burnout can stem from feelings of emotional exhaustion and disconnection from clients. Higher nurse-patient ratios can lead to an imbalance in work-life balance, work overload, and occupational hazards, all of which can result in personal, work-related, and client-related burnout.

Higher nurse-patient ratios can lead to an imbalance between work and personal life, work overload, and occupational hazards, all of which can contribute to personal, work-related, and client-related burnout. WSS, including workplace justice [40], supervisor support, and coworker support, can mitigate the effects of high nurse-patient ratios on burnout. A supportive work environment can provide a sense of belonging, recognition, and validation, leading to a lower likelihood of burnout. Taiwan is facing a shortage of healthcare workers due to an aging population, despite the implementation of the Long-Term Care Ten-Year Plan 2.0 [41]. Nurses are in short supply, and the nurse-patient ratio is higher than in other countries. Managers can reduce nursing burnout by investing in medical equipment and improving nurse-to-patient ratios [13].

In conclusion, to address nursing job burnout and improve patient care, healthcare managers should prioritize the implementation of WSS programs. These programs can help identify and address nurses' stressors, promote a positive working environment, encourage work-life balance, and mitigate job burnout. In addition, addressing the shortage of nurses through investment in medical equipment and an increase in nursing staff can help alleviate some of the burdens placed on nurses and improve the quality of patient care.

### 5.1. Limitations

Despite providing valuable insights into the connection between WSS, job stress, and nurse burnout, this study has several limitations that must be considered. First, the research was conducted at a single medical center in Taiwan, which may restrict the applicability of the findings to other healthcare environments. Additionally, the study's participants were limited to nurses working in a public medical center, excluding managers and those on unpaid leave, which could affect the representativeness of the sample. Lastly, the study relied on self-reported data, which might be influenced by response bias or the desire to present oneself in a socially desirable manner. Furthermore, as a cross-sectional study, it offers only a snapshot of a specific population at a given point in time, making the sequencing of events or establishment of causality problematic. The ambiguity regarding whether the exposure or outcome occurred first introduces an element of uncertainty. Consequently, one should proceed with caution when interpreting the study's results for inference purposes.

## 6. Conclusions

This research has provided a deeper understanding of the relationship between WSS, job stress, and burnout among nurses in Taiwan, focusing on the mediating role of WSS. The study significantly contributes to the existing body of knowledge and bridges both theoretical and practical gaps in the field of nursing burnout.

From a theoretical standpoint, this study contributes a new dimension to our understanding of WSS in the context of job stress and nurse burnout. The research findings highlight the crucial intermediary role of WSS in mitigating the impact of job stress on burnout. Before this research, such insights, especially within the Taiwanese healthcare setting, were limited. By exploring and highlighting the mediating role of WSS, this study addresses a theoretical gap in the existing literature. From a practical perspective, the study presents valuable implications for nursing management. It provides a clear roadmap for implementing strategies to alleviate nurse burnout and enhance the quality of patient care. The research suggests that enhancing WSS, promoting a positive work environment, facilitating work-life balance, and addressing nursing staff shortages could significantly reduce burnout levels. This practical insight allows healthcare administrators and nurse managers to devise effective strategies to mitigate burnout among their nursing personnel, thus addressing a crucial practical gap in the field.

This study enriches the theoretical understanding of the relationship between WSS, job stress, and nurse burnout and offers practical strategies for mitigating nurse burnout. It bridges the gap between what we previously understood about the subject and what we now understand, providing a solid foundation for future research and practical applications in this area.

## 7. Implications for Nursing Management

The results of this study offer numerous considerations for nursing management regarding the mitigation of nurse burnout and the enhancement of the quality of patient care. By identifying the elements contributing to job burnout and the crucial function of WSS, nurse managers and hospital administrators can devise and apply effective tactics to decrease burnout among nursing personnel. The following suggestions may direct nursing management in establishing a supportive work setting and improving the welfare of nursing professionals. First, nurse managers must endeavour to offer emotional, informational, and instrumental support to their nursing staff. This includes conducting routine evaluations of nurses' well-being, providing resources for stress management, and maintaining an adequate nursing workforce to avoid work overload. Furthermore, nursing management should promote and enable supportive connections between nurses and other healthcare practitioners. Second, nursing managers need to acknowledge the significance of work-life balance in averting burnout and fostering nurses' welfare. Managers can provide caregivers with work-life balance measures such as arranging a suitable workplace, providing for family resource needs (e.g., childcare), and fostering a culture that values personal time and self-care. Third, hospitals must provide sufficient nursing staff to address high nurse-patient ratios and workload overload. This entails investing in the recruitment and retention of skilled nursing personnel and offering training and development opportunities. Lastly, hospitals should consistently evaluate the efficacy of the strategies employed to alleviate job burnout. This can be achieved through surveys, feedback sessions, and the examination of key performance indicators related to staff welfare, job satisfaction, and turnover rates. In conclusion, nursing supervisors play an important role in decreasing nurses' burnout and promoting a positive practice environment in nursing, thereby improving healthcare quality and patient safety.

## Figures and Tables

**Table 1:** Distribution of demographic data and nursing burnout across categorical elements (*N* = 500).

Variables	*N* (%)	Personal burnout mean (SD)	Work-related burnout mean (SD)	Client-related burnout mean (SD)
Age (years)				
20–29	235 (47.0)	50.32 (16.80)	48.51 (17.04)	36.13 (17.44)
30–39	121 (24.2)	54.59 (17.50)	50.37 (17.30)	38.29 (15.54)
40–49	90 (18.0)	55.06 (18.35)	48.72 (15.52)	36.53 (13.94)
≥50	54 (10.8)	50.74 (21.24)	45.56 (20.85)	35.26 (17.43)
*t/F*		2.50	0.98	0.61
*p*		0.059	0.400	0.608
Gender				
Male	19 (3.8)	53.42 (18.71)	51.84 (17.97)	41.67 (17.62)
Female	481 (96.2)	52.20 (17.84)	48.56 (17.27)	36.43 (16.32)
*t/F*		0.09	0.66	1.87
*p*		0.771	0.417	0.172
Education level				
College	50 (10.0)	49.60 (16.84)	46.90 (17.55)	34.58 (15.55)
University	431 (86.2)	52.69 (17.65)	49.26 (17.04)	37.19 (16.54)
Masters or above	19 (3.8)	49.21 (24.17)	40.26 (20.44)	29.39 (13.21)
*t/F*		0.96	2.78	2.51
*p*		0.384	0.063	0.082
Marital status				
Single	326 (65.2)	51.67 (18.39)	49.48 (17.56)	37.32 (16.91)
Married	174 (34.8)	53.33 (16.79)	47.18 (16.71)	35.34 (15.32)
*t/F*		0.98	2.00	1.65
*p*		0.332	0.158	0.199
Working unit				
(1) Medical unit	128 (25.6)	49.73 (15.48)	47.50 (15.41)	37.79 (14.54)
(2) Surgical unit	112 (22.4)	51.92 (17.09)	47.59 (16.58)	35.75 (16.75)
(3) Gynecology and pediatrics	40 (8.0)	49.13 (19.74)	44.50 (16.82)	30.63 (13.98)
(4) General department ward	74 (14.8)	50.20 (18.50)	47.50 (18.38)	33.61 (17.60)
(5) ICU	101 (20.2)	57.92 (17.14)	52.82 (16.96)	37.42 (14.65)
(6) ER	31 (6.2)	55.48 (24.13)	55.32 (21.41)	49.46 (21.35)
(7) Dialysis room	14 (2.8)	49.64 (17.59)	41.79 (20.44)	32.14 (14.75)
*t/F*		2.78	2.78	5.10
*p*		0.012⁣^*∗*^	0.011⁣^*∗*^	0.000⁣^*∗∗∗*^
Scheffe		—	—	1, 2, 3, 4, 5, 7 < 6

*Note. *⁣^*∗*^*p* < 0.05, ⁣^*∗∗*^*p* < 0.01, ⁣^*∗∗∗*^*p* < 0.001.

**Table 2:** Distribution of demographic data and descriptive results of variables.

Variables	*N* (%)	Mean (SD)	Range
Age		34.65 (9.85)	20–58
Working years		9.11 (8.71)	0.25–31.67
<1 year	32 (6.40)		
1–5 years	235 (47.00)		
6–10 years	71 (14.20)		
11–20 years	85 (17.00)		
≥21 years	77 (15.40)		
Job stress		68.86 (9.92)	32–99
Workplace social support			
Workplace justice		63.30 (14.14)	0–100
Supervisor social support		11.97 (1.83)	4–16
Coworker social support		12.51 (1.74)	4–16
Job burnout			
Personal burnout		52.25 (17.85)	0–100
<80 points	464 (92.80)		
≥80 points	36 (7.20)		
Work-related burnout		48.68 (17.29)	0–100
<80 points	475 (95.00)		
≥80 points	25 (5.00)		
Client-related burnout		36.63 (16.39)	0–100
<65 points	476 (95.20)		
≥65 points	24 (4.80)		

*Note.* Personal burnout ≥80 points: severe; work-related burnout ≥80 points: severe; client related burnout ≥65 points: severe.

**Table 3:** Correlations among the continuous variables.

	WJ	SS	CS	OH	WLC	Overload	PB	WRB	CRB
WJ	1								
SS	0.669⁣^*∗∗*^	1							
CS	0.456⁣^*∗∗*^	0.543⁣^*∗∗*^	1						
OH	−0.231⁣^*∗∗*^	−0.192⁣^*∗∗*^	−0.110⁣^*∗∗*^	1					
WLC	−0.283⁣^*∗∗*^	−0.239⁣^*∗∗*^	−0.162⁣^*∗∗*^	0.632⁣^*∗∗*^	1				
Overload	−0.318⁣^*∗∗*^	−0.297⁣^*∗∗*^	−0.198⁣^*∗∗*^	0.610⁣^*∗∗*^	0.644⁣^*∗∗*^	1			
PB	−0.251⁣^*∗∗*^	−0.246⁣^*∗∗*^	−0.198⁣^*∗∗*^	0.430⁣^*∗∗*^	0.532⁣^*∗∗*^	0.429⁣^*∗∗*^	1		
WRB	−0.286⁣^*∗∗*^	−0.297⁣^*∗∗*^	−0.182⁣^*∗∗*^	0.437⁣^*∗∗*^	0.482⁣^*∗∗*^	0.428⁣^*∗∗*^	0.828⁣^*∗∗*^	1	
CRB	−0.215⁣^*∗∗*^	−0.202⁣^*∗∗*^	−0.153⁣^*∗∗*^	0.344⁣^*∗∗*^	0.344⁣^*∗∗*^	0.302⁣^*∗∗*^	0.505⁣^*∗∗*^	0.634⁣^*∗∗*^	1

*Note*. All correlations in this matrix were found to be statistically significant with a significance level of *p* < 0.01 (⁣^*∗∗*^). WJ, workplace justice; CS, coworker support; SS, supervisor support; OH, occupational hazards; WLC, work-life conflict; PB, personal burnout; WRB, work-related burnout; CRB, client-related burnout.

**Table 4:** Results of the unstandardized and standardized regression weights in the SEM.

Model pathways	Estimate^a^	S. E	C. R	*p*	Estimate^b^ (*β*)
NOSS ⟶ C-JCQ	−0.46	0.07	−6.80	<0.001	−0.38
C-JCQ ⟶ CBI	−0.20	0.06	−3.16	<0.001	−0.15
NOSS ⟶ CBI	0.89	0.09	10.16	<0.001	0.55
C-JCQ ⟶ WJ	1				0.77
C-JCQ ⟶ SS	0.70	0.05	14.81	<0.001	0.87
C-JCQ ⟶ CS	0.47	0.04	12.76	<0.001	0.61
NOSS ⟶ OH	1				0.77
NOSS ⟶ WLC	1.14	0.07	17.18	<0.001	0.83
NOSS ⟶ overload	1.07	0.06	16.61	<0.001	0.78
CBI ⟶ PB	1				0.86
CBI ⟶ WRB	1.09	0.04	25.40	<0.001	0.97
CBI ⟶ CRB	0.83	0.05	16.32	<0.001	0.65

*Note*. Outcomes were calibrated, taking into account factors such as age, gender, marital status, educational level, and unit. Estimate^a^, unstandardized regression weights, and Estimate^b^, standardized regression weights. C-JCQ, workplace social support; NOSS, nurses' stresses; CBI, nurse burnout; WJ, workplace justice; CS, coworker support; SS, supervisor support; OH, occupational hazards; WLC, work-life conflict; PB, personal burnout; WRB, work-related burnout; CRB, client-related burnout.

**Table 5:** Direct and indirect effects of NOSS on CBI.

	Path	Effect	Percentage of the total effect
Direct effects	NOSS ⟶ CBI	0.55	90.7
Indirect effects	NOSS ⟶ C-JCQ ⟶ CBI	−0.378 × (−0.149) = 0.06	9.3
Total effect		0.61	

*Note*. NOSS, nurses' stresses; CBI, nurse burnout; C-JCQ, workplace social support.

## Data Availability

The data used in this study are present within the article.
